# RNA-dependent RNA polymerase 1 in potato (*Solanum tuberosum*) and its relationship to other plant RNA-dependent RNA polymerases

**DOI:** 10.1038/srep23082

**Published:** 2016-03-16

**Authors:** Lydia J. R. Hunter, Samuel F. Brockington, Alex M. Murphy, Adrienne E. Pate, Kristina Gruden, Stuart A. MacFarlane, Peter Palukaitis, John P. Carr

**Affiliations:** 1 Department of Plant Sciences, University of Cambridge, Cambridge CB2 3EA, UK; 2 The James Hutton Institute, Invergowrie, Dundee DD2 5DA, UK; 3 National Institute of Biology, Večna pot 111, 1000 Ljubljana, Slovenia; 4 Division of Environmental and Life Sciences, Seoul Women’s University, Seoul, Republic of Korea

## Abstract

Cellular RNA-dependent RNA polymerases (RDRs) catalyze synthesis of double-stranded RNAs that can serve to initiate or amplify RNA silencing. *Arabidopsis thaliana* has six *RDR* genes; *RDRs 1*, *2* and *6* have roles in anti-viral RNA silencing. *RDR6* is constitutively expressed but *RDR1* expression is elevated following plant treatment with defensive phytohormones. RDR1 also contributes to basal virus resistance. RDR1 has been studied in several species including *A. thaliana*, tobacco (*Nicotiana tabacum*), *N. benthamiana*, *N. attenuata* and tomato (*Solanum lycopersicum*) but not to our knowledge in potato (*S. tuberosum*). *StRDR1* was identified and shown to be salicylic acid-responsive. *StRDR1* transcript accumulation decreased in transgenic potato plants constitutively expressing a hairpin construct and these plants were challenged with three viruses: potato virus Y, potato virus X, and tobacco mosaic virus. Suppression of *StRDR1* gene expression did not increase the susceptibility of potato to these viruses. Phylogenetic analysis of *RDR* genes present in potato and in a range of other plant species identified a new *RDR* gene family, not present in potato and found only in Rosids (but apparently lost in the Rosid *A. thaliana*) for which we propose the name *RDR7*.

RNA silencing is a set of mechanisms whereby gene expression is suppressed in a sequence-specific manner. In plants RNA silencing is initiated by cleavage of double-stranded (ds) RNA precursors into 21–24 nucleotide small RNA (sRNA) by DICER-LIKE (DCL) endonuclease^1^. Depending on the nature of the precursor molecules sRNAs are classified as microRNAs [miRNAs: imperfectly ds molecules derived from stem-loop structures in endogenous transcripts] or short-interfering (si)RNAs, which are perfectly complementary dsRNA molecules derived from ds structures occurring in a variety of cellular and foreign substrates, including virus-derived RNA molecules. Following methylation catalyzed by HUA ENHANCER1 (HEN1) and unwinding, single-stranded (ss) sRNAs are incorporated into RNA-induced silencing complexes (RISCs) in which they direct the sequence-specific activity of an ARGONAUTE (AGO) protein against nucleic acid sequences that are complementary to the sRNA. RISC activity may include RNA cleavage or translational inhibition of mRNA^1^,^2^,^3^,^4^,^5^,^6^,^7^,^8^, or the methylation of specific DNA sequences^9^,^10^,^11^.

In plants and certain other eukaryotes, RNA silencing can be amplified by the activity of cellular RNA-dependent RNA polymerases (RDRs)^12^,^13^,^14^,^15^. RDRs synthesize dsRNA from ssRNA templates by primer-independent and primer-dependent mechanisms^16^. Primer-dependent production of dsRNA requires primary siRNA in order to recruit the RDR and initiate synthesis. The dsRNA synthesized by RDR activity is in turn processed by DCLs into secondary siRNAs that feed back into the silencing pathway^17^,^18^,^19^.

RDRs are encoded by a small gene family and share a putative catalytic domain, the DLDGD motif^1^,^19^,^20^. Varying numbers of *RDR* genes with various biological effects have been identified in different plant species. For example, *Arabidopsis thaliana* (hereafter referred to as Arabidopsis) has six *RDR* genes (*RDR1*, *2, 3a, 3b, 3c* and *6*), rice has five and *Nicotiana benthamiana* has three^19^. In tomato, the resistance genes *Ty-1* and *Ty-3* encode RDRs that direct methylation-mediated inactivation of the DNA genomes of geminiviruses and these genes possess some sequence similarities to *AtRDRs 3a, 3b* and *3c*^21^. This suggests there is a degree of specialization or redundancy between different RDRs.

In Arabidopsis and a number of other plants, RDRs 1 and 6 have been shown to contribute to resistance against RNA viruses. RDR2, which is involved in establishment of transcriptional gene silencing, has also been implicated in anti-viral defence^12^,^17^,^22^,^23^,^24^,^25^,^26^,^27^,^28^,^29^,^30^,^31^. RDR6 is the best studied RDR with respect to its antiviral activities and it likely provides protection against the widest range of viruses. The other main ‘anti-viral’ RDR, RDR1, has stimulated considerable research because its expression is increased transiently by the defence-related signal molecules salicylic acid (SA), jasmonic acid (JA), ethylene, nitric oxide, as well as several other phytohormones, leading to suggestions that is involved not only in basal defence mechanisms but also in induced resistance mechanisms, such as systemic acquired resistance^22^,^23^,^25^,^32^,^33^,^34^,^35^. The importance of RDR1 in plant defence extends beyond resistance to viruses; RDR1 regulates JA-mediated changes in gene expression and defence against herbivory in *Nicotiana attenuata*^32^, and it influences expression of transcripts associated with pathogen resistance in tobacco (*N. tabacum*)^36^.

Potato (*Solanum tuberosum*) is the third most important food crop with an annual global production exceeding 374 million tonnes^37^. At least 40 viruses and two viroids are known to infect potato plants in the field, while a further 22 viruses and four viroids can infect potato plants experimentally^38^,^39^. However, the importance or otherwise of RDRs in potato-virus interactions is not well understood. In this paper we sought to explore the extent of the potato *RDR* gene family and investigate the role of StRDR1 in basal resistance to potato virus Y (PVY), potato virus X (PVX) and tobacco mosaic virus (TMV), which are three potato-infecting viruses for which RDR1 provides some level of protection in other hosts.

## Results

### Bioinformatic and phylogenetic analysis of plant RNA-dependent RNA polymerase genes

*RDR* genes with various biological effects have been identified in different plant species (summarized in Supplementary Table S1). Eight potential *StRDR* genes were identified by analysis of the Potato Genome Sequencing Consortium (PGSC) data. These include two *RDR1* genes, referred to as *StRDR1*a and *b* and three *RDR6* genes, referred to as *StRDR6a*, *b* and *c*. The *Solanum RDR3* genes are the product of recent duplications peculiar to the *Solanaceae* and the Arabidopsis *RDR3* genes are similarly recent and due to duplications in the *Brassicaceae*. As such there is no one-to-one orthology between the Arabidopsis *RDR3a*, *b*, and *c* genes (in earlier papers sometimes abbreviated as *AtRDR3*, *4*, and *5*, respectively: ref. 19) and the *Solanum RDR3* genes, although they do belong to the same orthogroup. To recognize this we refer to them here as *StRDR3e*, *d*, and *f* (Supplementary Fig. S1).

**Figure 1:**
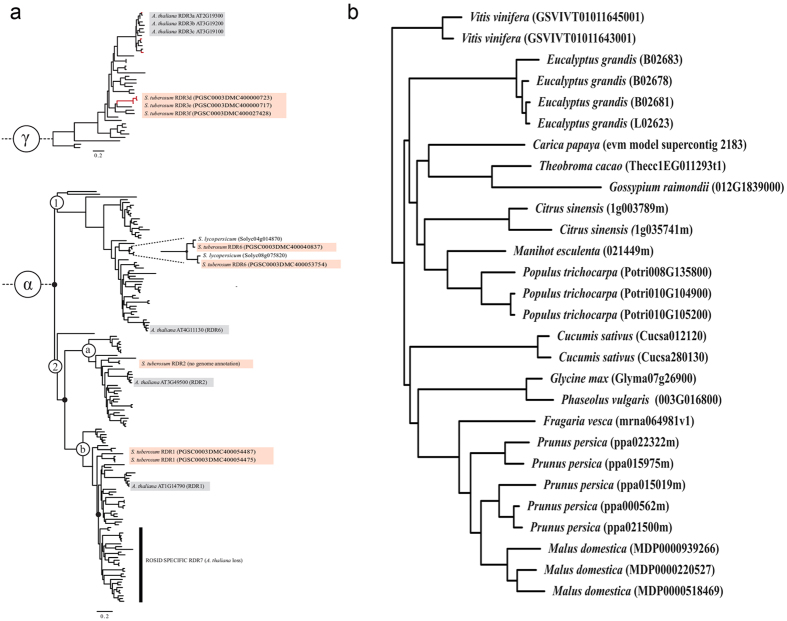
Phylogenetic analysis of plant *RDR* genes. (**a**) Phylogeny of the potato *RDR* genes with other plant *RDR*s. White open circles are clade names, black circles are inferred gene duplications, red boxes are *Solanum tuberosum* sequences and grey boxes are *Arabidopsis thaliana* sequences. The *S. tuberosum RDR* sequences found on the Potato Genome Sequencing Consortium (PGSC) database are accompanied by their PGSC coding-sequence number. (**b**) Rosid-specific *RDR7* phylogeny. This enlarged section of (**a**) shows the Rosid species that possess *RDR7* gene sequences.

The PGSC reference numbers identified for each *StRDR* gene locus, transcript, and coding sequence, as well as potential redundant and non-redundant *RDR* genes, are presented in Supplementary Fig. S1). Phylogenetic analysis of potato *RDR* sequences was performed to ensure that annotation of the potato *RDRs* by orthology assignment was to authenticated plant *RDRs*. It also allowed crosschecking for the presence or absence of a potato *RDR2*, for which no candidate sequence was initially apparent in the PGSC data. In Fig. 1a, the plant *RDR* phylogenetic tree that was produced using all published embryophyte plant genomes available in Phytozome v9 (http://www.phytozome.net/; date of access 17/09/2015) shows that most Arabidopsis and potato *RDRs* grouped according to the standard annotation^19^. Incorporating data from additional genetic resources such as the *Solanum* cDNA EST databases [NCBI BLAST (http://blast.ncbi.nlm.nih.gov/Blast.cgi#alnHdr_56775579; date of access 17/09/2015) and Phytozome (http://www.phytozome.net/; date of access 17/09/2015)] revealed the existence of a potato *RDR2* (Fig. 1a).

Figure 1a shows two separate groups of *RDR* genes, indicated by γ and α. This tree was constructed by separating these two groups because the Arabidopsis and potato *RDR3* genes (shown in γ) bear little sequence resemblance to each other or to other *RDR* genes and were essentially non-alignable. They were therefore analysed separately and, as referred to above, the three potato *RDR3* genes were referred to as *StRDR3e*, *d,* and *f*, to distinguish them from the Arabidopsis genes *AtRDR3 a*, *b* and *c* (*AtRDR3*, *4*, and *5*: ref. 19). Although these three Arabidopsis and potato *RDR3* genes fall into the γ lineage they are each the product of recent duplications. Thus, the potato *RDR3* genes are more closely related to each other than to the Arabidopsis *RDR* genes. Consequently, the potato *RDR3* genes have not been named according to an equivalent Arabidopsis *RDR* in this clade.

Analysis of the available genome data for potato showed that there are two *StRDR1* genes, which will be subsequently referred to as *StRDR1a* and *StRDR1b*. Alignment of the two transcribed *StRDR1* sequences demonstrated that both are 3,348 nt in length and differ only by 17 nt, which are spread throughout the length of the sequences. These nucleotide differences result in 11 amino acid differences at the protein level. An alignment between the two predicted protein sequences demonstrated that the differences between the two StRDR1 proteins do not affect regions of amino acid sequence conserved among RDRs or those characteristic of RDR1 proteins^19^,^40^. Therefore, although there are two *StRDR1* genes, comparison of the nucleotide and protein sequence alignments indicates they are equally likely to be functional *in vivo*. There is no evidence suggesting that either have been rendered non-functional due to a change in nucleotide sequence, such as, for example, the *RDR1m* gene that occurs in the widely used laboratory strain of *N. benthamiana*^41^, and which is a natural mutant due to a 72 nt insert within the open reading frame, containing tandem, in-frame stop codons^25^.

The apparent existence of three *RDR6* genes in potato (indicated in the PGSC data) was puzzling as in most other plant species examined to date only one *RDR6* gene sequence has been identified; barley (*Hordeum vulgare*) being the only known exception, with two *RDR6* genes^42^. However, our alignment of the three potato RDR6 protein sequences (data not shown) indicates that a sequencing error was included in the PGSC data and that the putative *StRDR6b* and *StRDR6c* sequences are two parts of the same *RDR6* locus. The alignment indicates that only one of these three RDR6 proteins (StRDR6a) is of full length (1,199 amino acids) and the other two proteins align roughly to either the N or the C termini of the full-length StRDR6 proteins (StRDR6b and StRDR6c, respectively). Furthermore, using the PGSC genome browser (http://solanaceae.plantbiology.msu.edu/cgi-bin/gbrowse/potato/; date of access 17/09/2015) it was found that the *StRDR6b* and *StRDR6c* loci are immediately adjacent to each other in the genome. Thus, potato has two *RDR6* genes, here designated as *RDR6a*, and *RDR6b.* Figure 1a shows that tomato also has two *RDR6* genes. This was confirmed also when checking the second available gene model produced by the International Tomato Genome Sequencing Project consortium: https://solgenomics.net/organism/Solanum_lycopersicum/genome; date of access 17/09/2015. This implies that *RDR6* gene duplication occurred during the evolution of the genus *Solanum* and hence this is not specific to potato.

### Identification of a novel Rosid-specific member of the *RDR* gene family: *RDR7*

Phylogenetic analyses were extended to include genomic data from a number of species in which *RDR* genes had not been examined previously. This resulted in the discovery of a previously unknown *RDR* clade, which we named *RDR7* (Fig. 1b). *RDR7* is Rosid-specific and although Arabidopsis is a Rosid^43^, *RDR7* does not appear to exist in Arabidopsis, suggesting that *RDR7* has been lost in this lineage (Fig. 1a). Potato is an Asterid and does not have an *RDR7*. Supplementary Table S1 shows in which plant species *RDR* genes have been studied and illustrates that the majority of research has been done on Arabidopsis as well as *Nicotiana* species, which are Asterids. Therefore, it is perhaps not surprising that *RDR7* has remained unnoticed. Rosid species that were found to have *RDR7* are shown in Fig. 1b, which presents the *RDR7* region from the *RDR* phylogeny. These include a number of well-known food crops such as cassava (*Manihot esculenta*), common bean (*Phaseolus vulgaris*), soybean (*Glycine max*), cucumber (*Cucumis sativus*), cocoa (*Theobroma cacao*), papaya (*Carica papaya*), apple (*Malus domestica*), strawberry (*Fragaria vesca*), grape (*Vitis vinifera*), and orange (*Citrus sinensis*), as well as tree species such as *Populus trichocarpa* and eucalyptus (*Eucalyptus grandis*) (Fig. 1b).

Two Rosid species identified as having *RDR7* genes, peach (*Prunus persica*) and cotton (Supplementary Table S1), have been used in previous research on RDRs by Di Serio *et al.*^44^ and Wang and co-workers^45^, respectively, but neither earlier study noted the existence of a Rosid-specific *RDR* gene. However, characterization of peach *RDR* genes was not the focus of the paper by Di Serio and colleagues^44^ and the cotton species (*Gossypium hirsutum*) studied by Wang and co-workers^45^ was different to the cotton species (*G. raimondii*) identified in our phylogenetic analysis (Fig. 1b).

### Salicylic acid enhances accumulation of potato *RDR1* transcripts

**Figure 2:**
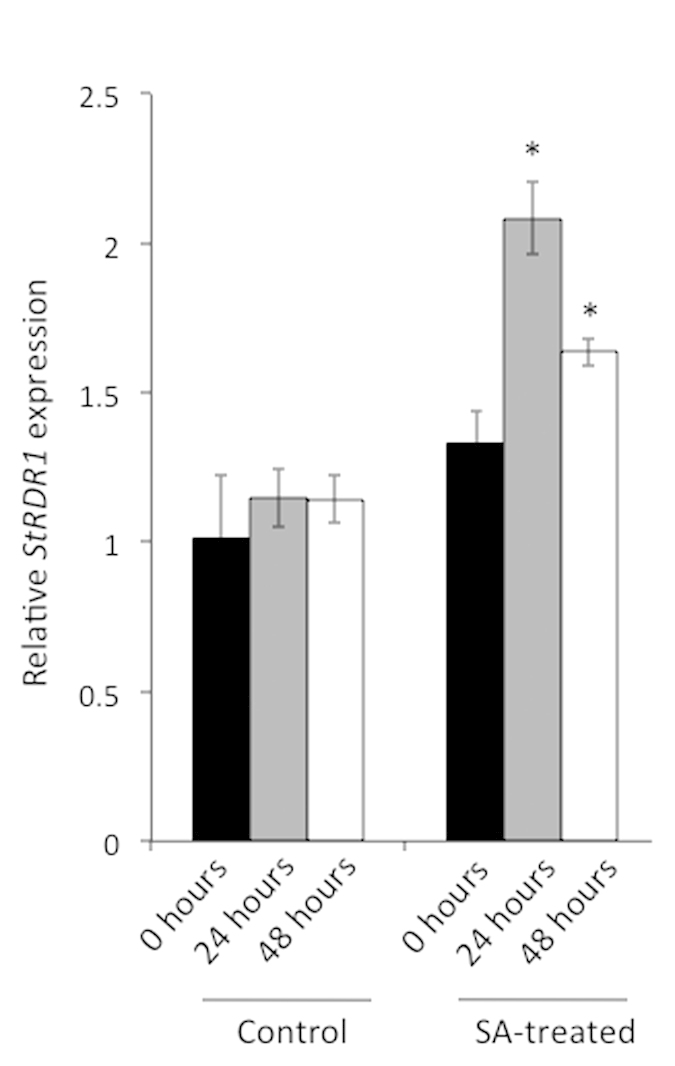
RTqPCR analysis of *StRDR1* expression after salicylic acid (SA) treatment of Pentland Dell potato plants. Samples were taken at 0 hours (immediately before treatment), 24 hours and 48 hours after SA or control (water) treatment. Control-treated Pentland Dell plants were sprayed with water and the control sample at 0 hours was used as the reference. Cyclophilin was used as the reference gene for the RTqPCR analysis. Significant increases in *StRDR1* transcript accumulation were seen at 24 hours and 48 hours after SA treatment (p < 0.005; *). Error bars represent standard error of the mean.

SA increases *RDR1* gene expression in *Nicotiana* species and Arabidopsis^22^,^25^,^33^. To investigate whether potato *RDR1* expression also responds to this phytohormone, potato plants (cv. Pentland Dell) were treated with 1 mM SA and *StRDR1* expression was analysed by a reverse transcription coupled to the quantitative polymerase reaction (RTqPCR) using appropriate primers designed to allow detection of both *StRDR1a* and *StRDR1b*. Figure 2 shows relative *StRDR1* transcript accumulation in control (water-treated) and SA-treated leaves at 0 (immediately before treatment), 24 and 48 hours after treatment. SA-treated leaves show a significant two-fold increase in *StRDR1* expression at 24 hours post treatment in comparison to the control treatment at 24 hours. By 48 hours post treatment, *StRDR1* expression had declined but was still significantly higher than in control-treated plants at that time point. This experiment was done three times with similar results.

### Depletion of *StRDR1* transcript accumulation in transgenic potato

**Figure 3:**
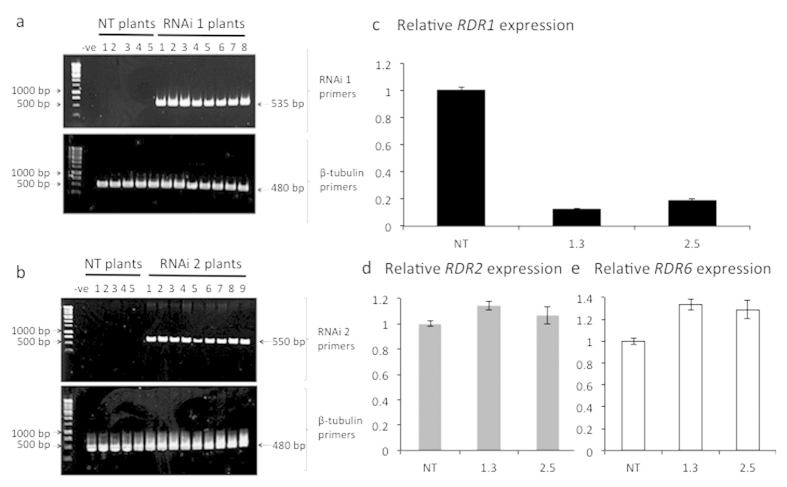
Generation of *StRDR1*-depleted transgenic potato lines. PCR of plant genomic DNA to verify the presence of the *StRDR1*-specific hairpin constructs RNAi1 (**a**) or RNAi2 (**b**) in the transformed potato lines. PCR used a primer complementary to a sequence located in the 35S promoter of the T-DNA insert and primers specific to either RNAi 1 and 2, to amplify regions of 535 bp and 550 bp, respectively. DNA from non-transformed (NT) plants and a no template PCR control (negative: -ve) were included in the analysis and control PCR reactions were carried out using primers specific for β-tubulin (expected product of 480 bp). (**c**) *StRDR1* accumulation is efficiently decreased in a potato lines expressing the RNAi1 (Line 1.3) and RNAi2 construct (Line 2.5) (for other examples see Supplementary Fig. S3). Accumulation levels of transcripts of *StRDR2* (**d**) and *StRDR6* (**e**) were not diminished in plants of transgenic lines expressing either RNAi1 or RNAi2 to inhibit StRDR1 expression. Error bars represent standard error of the mean.

Two DNA constructs encoding dsRNA hairpin transcripts were designed to silence the transcripts of *StRDR1a* and *StRDR1b* (Supplementary Table S2). Construct 1 corresponded to a sequence in the 5′ region of the *StRDR1* transcripts and the other (Construct 2) to a sequence in the 3′ region (Supplementary Fig. S2). A BLAST search using the two chosen RNA interference (RNAi) inducing sequences was done to ensure they did not match other plant gene sequences. Transgenic potato (cv. Pentland Dell) lines expressing either of the two hairpin constructs (Supplementary Fig. S2) were made and lines showing decreased accumulation of *StRDR1* transcripts were identified (Fig. 3 and Supplementary Fig. S3). Both hairpin constructs appeared to be equally effective at diminishing accumulation of *StRDR1* transcripts. Therefore, in most subsequent experiments to assess the effect of silencing *StRDR1* on the susceptibility of potato plants to viral disease, line 1.3 (expressing Construct 1) was used. Figure 3c shows the relative *RDR1* expression for lines 1.3 and 2.5 in comparison to non-transformed plants. To ensure that there were no off-target effects of either of the RNAi constructs, the accumulation of *StRDR2* (Fig. 3d) and *StRDR6* (Fig. 3e) transcripts were compared in non-transformed plants and transgenic lines 1.3 and 2.5. It was found that expression of silencing constructs for *StRDR1* did not affect the expression of the *StRDR2* or the *StRDR6* genes.

### Down-regulation of *StRDR1* expression did not enhance susceptibility to tobacco mosaic virus

**Figure 4:**
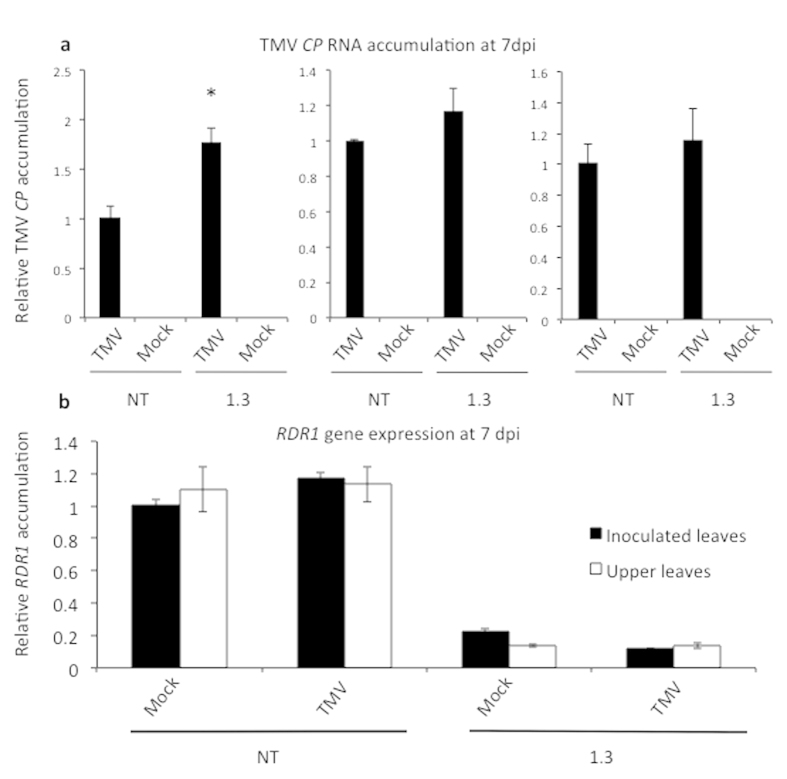
TMV infection and *StRDR1* transcript accumulation in *StRDR1*-depleted transgenic potato plants. (**a**) RTqPCR was used to determine the relative level of TMV coat protein (*CP*) RNA (**a**) in inoculated leaves of non-transformed (NT) and transgenic Line 1.3 plants at 7 days post-inoculation (dpi). RNA was also extracted from mock-inoculated leaves. In each of three independent experiments, TMV *CP* RNA accumulation in transgenic samples was normalized to that in the non-transformed control. In only one experiment was any significant increase in CP accumulation noted (p = 0.007: *) (**b**) RTqPCR was used to measure accumulation, relative to that mock-inoculated non-transformed plant, of *RDR1* in mock-inoculated and TMV-inoculated plants at 7 dpi in inoculated, mock-inoculated leaves and upper non-inoculated leaves. Error bars represent standard error of the mean.

TMV has been used as the model virus in several previous studies of RDR1 in solanaceous hosts (see introduction). Non-transgenic plants of the potato cultivar Pentland Dell were susceptible to TMV infection in directly-inoculated leaves but the virus did not spread systemically. Figure 4a shows relative TMV RNA accumulation at 7 days post inoculation (dpi) in inoculated leaves of non-transformed and *StRDR1*-silenced line 1.3 in three independent experiments. TMV RNA accumulated to similar levels in the inoculated leaves of non-transformed plants and transgenic plants with diminished *StRDR1* transcript accumulation. In only one out of three experiments was any increase noted in the accumulation of TMV RNA in transgenic plant tissue compared to non-transformed (Fig. 4a left panel: *p *= 0.007). TMV RNA could not be detected in the upper, non-inoculated leaves of either non-transformed or transgenic plants and infection with TMV did not appear to result in the appearance of disease symptoms in either type of plant. Increased *RDR1* expression has been proposed to be associated with TMV infection in susceptible tobacco^22^. However, we did not see any increase in *StRDR1* transcript accumulation following infection of non-transformed or transgenic potato plants with this virus (Fig. 4b).

### Down-regulation of *StRDR1* expression did not diminish resistance to PVX or PVY^O^

We examined the effect of depletion of *StRDR1* transcript accumulation on the ability of resistant potato plants to inhibit virus spread. PVX induces visible necrotic hypersensitive response (HR) lesions at inoculation sites in the cultivar Pentland Dell due to the presence of the resistance genes, *Nx* and *Nb*^39^,^46^. PVY^O^ causes severe disease symptoms in susceptible potato cultivars but in Pentland Dell the virus triggers visible HR lesions but does not normally spread systemically. We examined, in plants with diminished *StRDR1* transcript accumulation, if either virus was able to escape the confinement imposed by HR-type resistance or replicate to higher levels than in non-transformed plants.

**Figure 5:**
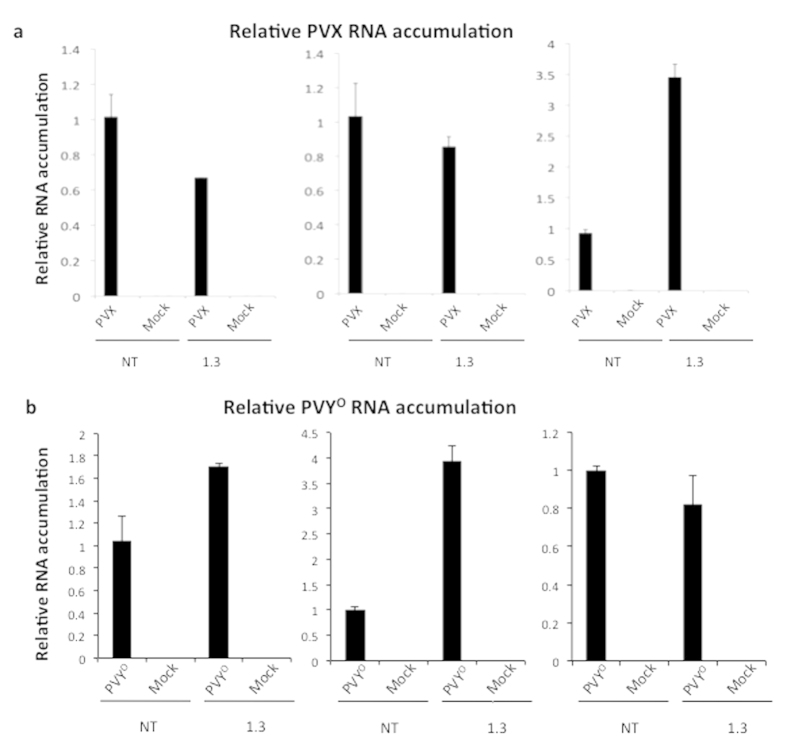
Accumulation of PVX and PVY^O^ RNA in *StRDR1*-depleted transgenic potato plants. RTqPCR was used to determine the relative levels of PVX (**a**) and PVY^O^ (**b**) RNA in inoculated leaves of non-transformed (NT) and transgenic Line 1.3 plants at 7 and 10 days post-inoculation, respectively. RNA was also extracted from mock-inoculated leaves. In each of three independent experiments, viral RNA accumulation in transgenic samples was normalized to that in the non-transformed control. Error bars represent standard error of the mean.

Using primers specific for either PVX or PVY^O^ viral RNA accumulation was assessed by RTqPCR in inoculated leaves of non-transformed plants and plants of the *StRDR1*-depleted line 1.3 (Fig. 5). Three independent experiments were carried out for each virus but neither PVX nor PVY^O^ showed any consistent increase in accumulation in the inoculated leaves of transgenic line 1.3 versus the leaves of the non-transformed controls (Fig. 5). Although there was variation in the relative viral RNA accumulation in inoculated leaves of plants of transgenic line 1.3 (see Fig. 5a, right panel and Fig. 5b, middle panel), it is likely to have resulted from variation in the numbers of inoculation sites per leaf. There were no obvious changes in HR lesion morphology on leaves of transgenic line 1.3 inoculated with PVX or PVY^O^ (Supplementary Fig. S4) and no symptoms were apparent on the upper, non-inoculated leaves of these plants (Supplementary Fig. S5). Attempts to detect RNA of either virus in non-inoculated leaves of transgenic line 1.3 plants were unsuccessful. Therefore, silencing *StRDR1* did not result in any breakdown in HR-type resistance to PVX or PVY^O^.

Since *StRDR1* is SA-responsive and since SA is produced at increased levels following an HR in other solanaceous hosts, particularly in the vicinity of the lesion^47^,^48^, we investigated whether *StRDR1* transcript accumulation was increased in leaves of non-transformed or *StRDR1*-depleted transgenic plants following inoculation with either PVX or PVY^O^. However, no increase in *StRDR1* expression was detectable in these tissues (Supplementary Fig. S6).

## Discussion

Prior to designing constructs to down-regulate *StRDR1* expression in potato we carried out bioinformatic and phylogenetic analyses of the *RDR* gene family in this plant. Initially, this was carried out to aid the design of silencing constructs. However, we extended our analyses to encompass a range of other plant species to obtain a broader insight into the evolution of *RDR* genes. We found that potato has eight *RDR* genes in total, which were named *StRDR1a, StRDR1b, StRDR2, StRDR3d, StRDR3e, StRDR3f*, *StRDR6a* and *StRDR6b* (Fig. 1a). The naming of *StRDR3* family members as *StRDR3d, StRDR3e, StRDR3f* was used since there is no direct orthology with the Arabidopsis genes *AtRDR3a*, *b*, or *c*. Construction of a phylogenetic tree indicated that the duplication of *StRDR6* is not specific to *S. tuberosum*, as *S. lycopersicum* also has two *RDR6* genes. Eight *RDR* genes is the largest complement of *RDR* genes possessed by any plant species examined to date. Until now, Arabidopsis was thought to have the highest number of *RDR* genes – six^19^. Our wider phylogenetic analysis identified a novel *RDR* clade. We have tentatively named this *RDR7* to distinguish it from *RDR1* since the *RDR1* and *RDR7* lineages split from each other approximately 10^8^ years ago and because *RDR7* is widely conserved across Rosid species but, does not occur in non-Rosid species (Fig. 1b). *RDR7* appears to have been lost during the evolution of Arabidopsis, the best studied Rosid species (Supplementary Table S1), and as a consequence it appears that *RDR7* has been overlooked in previous studies of *RDR* genes. To comment further on the biological function(s) of RDR7 or the evolution of the *RDR7* clade would be speculative at this stage since further research needs to be done to characterize the properties of this gene and its product.

A key characteristic shared by *RDR1* genes studied to date has been their transcriptional response to SA, as well as to a number of other phytohormones^22^,^23^,^25^,^32^,^33^,^34^. Consistent with previous studies, we found that SA treatment increased *StRDR1* transcript accumulation, which indicates that regulation of *RDR1* by this phytohormone is conserved in potato. *RDR1* expression has been reported to increase following tobamovirus infection in susceptible tobacco and Arabidopsis plants^22^,^23^ although this was likely an artefact of the wounding associated with mechanical inoculation in those plants^33^. We found that no increase in *StRDR1* transcript levels was detectable in either inoculated or non-inoculated leaves of potato plants in response to infection with TMV, indicating that *RDR1* transcript accumulation in potato is less sensitive to wounding, consistent with results indicating that *StRDR1* was not responsive to chewing by the Colorado potato beetle^49^. Although HR induction is associated with increased SA biosynthesis there was no evidence found for increased *StRDR1* transcript accumulation following elicitation of necrotic HR lesions by either PVX or PVY^O^.

We successfully down-regulated the levels of the transcripts for the potato *RDR1* genes (*StRDR1a* and *StRDR1b*) by expression of either of two hairpin constructs in transgenic plants. However, this did not cause any consistent or reproducible increase in susceptibility to TMV or breakdown of resistance to PVY^O^ or PVX in the inoculated leaves. These results were surprising in light of multiple previous reports that have demonstrated that RDR1 is involved in defence against viruses: TMV and tobacco rattle virus in Arabidopsis^23^,^28^; TMV, PVX and PVY in tobacco^22^,^36^, and against TMV, as well as two other tobamoviruses (turnip vein-clearing virus and sunn-hemp mosaic virus) in *N. benthamiana*^25^,^50^. These results suggest either that RDR1 is not as critical for antiviral defence in potato as it appears to be in other species, or (in the case of resistance to PVX and PVY on this host) that RDR1 is not critical for virus restriction during the HR, and in the case of TMV, its inability to escape from the inoculated leaves may be due to factors other than RDR1.

One explanation for these results might be that there is redundancy between potato RDRs. For example, StRDR6a and b might compensate for decreased levels of StRDR1a and b. Alternatively, StRDR1a and StRDR1b may be involved in processes other than antiviral defence, such as the regulation of endogenous mRNA, which appears to be one of the roles for RDR1 in *N. attenuata* and tobacco^32^,^36^. Given that there are eight *RDR* genes in potato, there may have been more scope during the evolution of this plant for RDR diversification and specialization than in other species. Thus, we speculate that RDRs 1a and 1b may have lost their roles in antiviral resistance (while retaining other roles) without any loss in plant fitness since the expression of RDRs 6a and 6b may be adequate for the antiviral defence needs of potato.

## Methods

### Bioinformatics and Phylogenetic analysis of plant *RDR* genes

Preliminary identification of putative RDR protein or nucleic acid sequences in potato, Arabidopsis, tobacco, tomato and other plant species was done by searching TAIR (http://www.arabidopsis.org/; date of access 17/09/2015), Swissprot (http://web.expasy.org/docs/swiss-prot_guideline.html; date of access 17/09/2015), Potato Genome Sequencing Consortium (PGSC)^51^ (http://www.potatogenome.net; date of access 17/09/2015), the Tomato Genome Sequencing Consortium^52^ (https://solgenomics.net/organism/Solanum_lycopersicum/genome; date of access 17/09/2015), and GoMapMan^53^ (http://www.gomapman.org/; date of access 17/09/2015). The PGSC sequence data used were obtained from a doubled monoploid (DM) potato clone from *Solanum tuberosum*, group *phureja*^51^. To identify all relevant potato gene sequences a non-stringent basic local alignment search tool (BLAST: ref. 54) search was run against the PGSC double monoploid gene/loci (DMg) data, by querying with Arabidopsis *RDR* sequences. Putative *RDR* sequences were downloaded in FASTA format. Transcript and protein sequences were identified for each of the potato genes by running another BLAST search against DM transcript (DMt), DM coding sequence (DMc), and DM protein (DMp) data accessible through the potato genome browser. These sequences were downloaded for each of the potato *RDR* genes for use in alignments and the DMt, DMc and DMp identifiers were entered into a spreadsheet next to their corresponding *RDR1* gene. Genes were classified into ‘gene families’ according to sequence similarity by checking E-values obtained from BLAST results when one DMg retrieves the other DMg sequences. Alignments of the DMc, non-redundant DMp, and Arabidopsis *RDR* sequences of interest were done using CLC Sequence Viewer (Qiagen, Hilden, Germany).

Putative *RDR* gene sequences were also obtained using BLAST search of Phytozome^55^ (http://www.phytozome.net; date of access 17/09/2015) against known orthogroups. The search string employed was the Arabidopsis *RDR1* sequence. Amino acid alignments were done with MAFFT^56^ (http://mafft.cbrc.jp/alignment/software/; date of access 17/09/2015) using an E-INS-I alignment strategy. Alignments were refined with Se-Al^57^ (http://tree.bio.ed.ac.uk/software/seal/; date of access 17/09/2015) and converted to corresponding aligned DNA sequences with RevTrans^58^ (http://www.cbs.dtu.dk/services/RevTrans/; date of access 17/09/2015). All phylogenetic analyses were conducted on nucleotide alignments. For maximum likelihood analyses the program GARLI (Genetic Algorithm for Rapid Likelihood Inference; version 0.942) was employed. GARLI conducts maximum likelihood heuristic phylogenetic searches under the GTR (generalized time reversible) model of nucleotide substitution, in addition to using models that incorporate among-site rate variation, either assuming a gamma distribution (Γ) or a proportion of invariable sites (I), or both. Analyses were run with default options, except that the “significanttopochange” parameter was reduced to 0.01 to make searches more stringent. A strict consensus of five replicate GARLI analyses was done, selecting the topology with the highest likelihood score.

### Potato plant growth conditions and treatments

Lines of non-transformed and transformed potato (*Solanum tuberosum* L.) plants of cultivar Pentland Dell were maintained by micro-propagation in tissue culture under sterile conditions. Potato plants were maintained on MS30 agar (Murashige and Skoog basal salts supplemented with 3% (w/v) sucrose and 1% (w/v) agar, adjusted to pH5.8). Micro-propagated lines were passaged using 1 cm stem pieces that included a petiole. Tissue cultured plant material was maintained at 22 °C and 16 hours of light.

Plants were also grown in Levington M3 compost (Scotts, Ipswich UK) and medium vermiculite (Scotts) at a ratio of 5:1, respectively with 0.002% (w/v) ‘Intercept 5GR’ (Scotts) either in a glasshouse (with supplementary lighting in winter), or in a growth chamber (Conviron Ltd., Winnipeg, Manitoba, Canada) under 16 hours of light, 200 μE.m^−2^.s^−1^, 22 °C, 60% humidity). To maintain the lines, cuttings were taken every four weeks by removing the apical 10 cm of the plant (cut below a node), dipped in rooting powder and planted in fresh compost mix.

For treatment with SA, plants were sprayed with 1 mM SA^33^. Potato plants were infected with PVY strain O (PVY^O^) by mechanical inoculation of two lower leaves with Carborundum as an abrasive using sap extracted from two systemically infected leaves of PVY-infected *Nicotiana benthamiana* plants in 0.8 ml of 0.1 M potassium phosphate buffer pH 7.2^59^. Mechanical inoculation with PVX strain UK3^60^ and TMV strain U1 used purified virions diluted to 50 μg/ml in potassium phosphate buffer^61^. Mock-inoculation used phosphate buffer only. Four plants were used for each treatment and experiments were carried out at least three times.

### Transformation constructs

The silencing construct design was based on that used previously in tobacco for silencing *NtRDR1* transcripts by Rakhshandehroo and colleagues^36^ and two target sequences (Supplementary Fig. S2) were chosen by lining up known *RDR1* gene sequences. DNA for the silencing constructs was amplified from potato DNA by PCR using Phusion DNA polymerase (Invitrogen, Waltham MA). Primers used for amplifying the first and second RNAi constructs are listed in Supplementary Table S2 (primer numbers 1–4). The Gateway cloning entry vector pDONR 207 (Invitrogen) was used to generate entry clones for each of the two RNAi constructs, following the manufacturers instructions. For RNAi sequences inserted into pDONR 207 plasmid, the Gateway LR reaction (Invitrogen) was used to recombine the construct sequences into the destination vector pK7GWIWG2(II)0 used for potato plant transformation^62^.

### Transformation

Micro-propagated Pentland Dell plants were used to obtain leaf and stem pieces for transformation. Stem sections (*c.* 5 mm in length) excised from inter-nodal regions were transferred to liquid Murashige and Skoog basal salts pH 5.8 containing 2% sucrose for co-cultivation with *Agrobacterium tumefaciens* LBA4404. *A. tumefaciens* cells harboring either Construct 1 or 2 were incubated in liquid LB medium^58^ at 28 °C for 48 hours prior to use in plant transformation. Co-cultivation was carried out at room temperature with gentle agitation at 25 rpm for 20 minutes. Stem pieces were transferred to half-strength MS30 medium amended with 0.2 mg/l naphthalene acetic acid, 2.5 mg/l zeatin riboside and 2 mg/l gibberellic acid for 48 hours, then transferred to the same medium with the addition of 250 μg/ml cefotaxime and 40 μg/ml kanamycin. Plant pieces were transferred onto fresh medium every 7 days for 4–5 weeks until callus had developed. Calli were transferred onto MS30 agar amended with 0.02 mg/l naphthalene acetic acid, 2 mg/l zeatin riboside, 2 mg/l gibberellic acid, 250 μg/ml cefotaxime and 40 μg/ml kanamycin, with weekly transfers. Shoots that developed from calli were transferred to MS30 agar amended 250 μg/ml cefotaxime and 40 μg/ml kanamycin to promote root formation.

Genomic DNA was extracted using the method of Doyle and Doyle^63^ from young leaves of transformed and non-transformed plants and PCR with primers designed to amplify regions within the RNAi constructs used to verify transgene insertion into the plant genome (primers 5–7 in Supplementary Table S2). Identification of potato lines transformed with the empty destination vector was achieved through PCR of genomic DNA from the empty vector potato line. The PCR primers used were situated either side of one of the insert regions on the destination vector, one in the 35S promoter (primer 5) and the other in the intron (primer 8).

Twelve independent transformed lines were chosen for RTqPCR analysis to assess down-regulation of *StRDR1* transcript expression. One plant from each independent line was sampled. Reverse transcription PCR and first-strand cDNA synthesis was done according to the manufacturers instructions. RTqPCR using Sybr Green (Sigma) was used to assess the degree of silencing in transformed and non-transformed lines (non-transformed lines were used as the reference samples). *Cyclophilin* was used as the reference transcript (primers 9 and 10 in Supplementary Table S2) as its expression was identified as being stable under the experimental conditions and virus-inoculated non-transformed plants were used as the reference samples during RTqPCR analysis. RTqPCR procedures and equipment used have been described previously^33^. *StRDR1*-specific RTqPCR primers were used to amplify the region of the *StRDR1* gene that lies between the regions of the *RDR1* gene that are included in two RNAi constructs (primers 11 and 12 listed in Supplementary Table S2). Primers were designed with sequences matching both *StRDR1a* and *StRDR1b*, so that *RDR1* expression analysis accounted for both of the potato *RDR1* genes.

### Analysis of viral infection of *RDR1*-silenced and non-transformed potato plants

During sampling, leaves were first photographed and then frozen in liquid nitrogen. Harvested leaves of the same potato line/treatment were pooled and stored at −80 °C prior to RNA extraction and RTqPCR analysis to assess relative virus accumulation. Both inoculated leaf tissue and upper, younger leaves were harvested for RNA extraction. Upper, non-inoculated leaves were sampled to assess whether silencing of *RDR1* might allow systemic viral movement. RTqPCR primers were designed to amplify the coat proteins of each of the viruses used, PVX, PVY^O^ and TMV (primers 13–18 in Supplementary Table S2). RTqPCR was performed and results analysed as previously described^33^.

## Additional Information

**How to cite this article**: Hunter, L. J. R. *et al.* RNA-dependent RNA polymerase 1 in potato (*Solanum tuberosum*) and its relationship to other plant RNA-dependent RNA polymerases. *Sci. Rep.* **6**, 23082; doi: 10.1038/srep23082 (2016).

## Supplementary Material

Supplementary Information
