# Supplementary material for: RNA-dependent RNA polymerase 1 in potato (Solanum tuberosum) and its relationship to other plant RNA-dependent RNA polymerases
Source: Sci Rep. 2016 Mar 16;6:23082. doi: 10.1038/srep23082 (PMC4793286; doi:10.1038/srep23082)
Supplement: Supplementary Information [file srep23082-s1.pdf]

# Supplementary Information

## RNA-dependent RNA polymerase 1 in potato (*Solanum tuberosum*) and its relationship to other plant RNA-dependent RNA polymerases

Lydia J. R. Hunter[1,2], Samuel F. Brockington[1], Alex M. Murphy[1], Adrienne E. Pate[1], Kristina Gruden[3], Stuart A. MacFarlane[2], Peter Palukaitis[2,4], John P. Carr[1]

1. Department of Plant Sciences, University of Cambridge, Cambridge CB2 3EA, U.K.

2. The James Hutton Institute, Invergowrie, Dundee DD2 5DA, U.K.

3. National Institute of Biology, Vecna pot 111, 1000 Ljubljana, Slovenia

4. Division of Environmental and Life Sciences, Seoul Women's University, Seoul, Republic of Korea

Correspondence and requests for materials should be addressed to J.P.C. (jpc1005@hermes.cam.ac.uk)

**Supplementary Table S1.** Known roles and characteristics of plants RDRs

| Plant species | RDR | Function | References |
|---|---|---|---|
| ***Arabidopsis thaliana*** | **RDR1** | **Antiviral silencing against:** | |
| | | Tobacco mosaic virus (TMV) and tobacco rattle virus (TRV) | **1** |
| | | TRV (combined activity of AtRDR1 with 2 and 6) | **2** |
| | | TMV | **3** |
| | | Turnip mosaic virus (TuMV) | **4** |
| | | Cucumber mosaic virus (CMV) | **5** |
| | **RDR6** | **Post-transcriptional gene silencing** | **6** |
| | | **Antiviral silencing against:** | |
| | | TRV (dependent on combined activity with AtRDR1 and 2) | **2** |
| | | TMV | **3** |
| | | TuMV | **4** |
| | | CMV | **5,7** |
| | | Cabbage leaf curl virus (CaLCuV: geminivirus, DNA genome) | **8** |
| | | ***ta*-siRNA pathway:** | |
| | | Component of the pathway | **9** |
| | | Regulation of shoot development | **10,11** |
| | | Regulation of leaf development | **12,13** |
| | | Regulation of lateral root development | **14** |
| | | Regulation of developmental timing and patterning | **15** |
| | | **nat-siRNA pathway:** | |
| | | Regulation of salt tolerance | **16** |
| | | Anti-bacterial defence against *Pseudomonas syringae* | **17** |
| | **RDR2** | **RdDM pathway:** | |
| | | Component of the pathway | **18,19,20,21,22** |
| | | Silencing signal between cells | **23** |
| | | Long-distance silencing signal | **24** |
| ***Nicotiana tabacum* (tobacco)** | **RDR1** | **Defence against:** | |
| | | TMV and potato virus X (PVX) | **25** |
| | | Potato virus Y (PVY) (and regulation of defence-related genes) | **26** |
| ***Nicotiana benthamiana*** | **RDR1** | **NbRDR1m is non-functional** | |
| | | - Transformation with the *Medicago truncatula RDR1* increased resistance to the tobamoviruses TMV, turnip vein clearing virus and sunn-hemp mosaic virus | **27** |
| | | - Transformation with the *N. tabacum RDR1* increased susceptibility to plum pox virus (PPV) | **28** |
| | | - The non-functional RDR1m does not occur in most wild accessions | **29** |

| | | | |
|---|---|---|---|
| | | of *N. benthamiana*, which possess functional RDR1 enzymes | |
| | **RDR6** | **Required for defence against:**<br>PVX, PVY and CMV<br>PVX, TMV and turnip crinkle virus (TCV)<br>PVX and PPV<br>**Required for pathogenesis induced by:**<br>Potato spindle viroid<br>Hop stunt viroid | <br>**30**<br>**31**<br>**32,33**<br><br>**34**<br>**35** |
| ***Nicotiana attenuata*** | **RDR1** | **Resistance to herbivorous insects:**<br>Through regulation of endogenous small RNAs | <br>**36,37** |
| | **RDR2** | **Regulation of resistance to UV-B:**<br>Through regulation of endogenous small RNAs | <br>**38** |
| | **RDR6** | **Antiviral silencing:**<br>Defence against TMV and bell pepper mosaic virus | <br>**39** |
| ***Nicotiana glutinosa*** | **RDR1** | Expression reported to be increased by salicylic acid, jasmonates, PVY, TMV, CMV and $H_2O_2$ | **40** |
| | **RDR6** | Expression reported to be affected by jasmonates, gibberellic acid (GA), abscisic acid (ABA), CMV and fungal infection | **41** |
| ***Solanum tuberosum* (potato)** | **RDR2** | **RdDM pathway:** Silencing of miniature inverted-repeat transposable elements (MITEs) | **42** |
| ***Solanum lycopersicum*** (syn. ***Lycopersicon esculentum***) **(tomato)** | **RDR1** | **First isolation and characterization of a plant RDR** | **43** |
| ***Solanum chilense*** | ***Ty-1***<br>***Ty-3*** | **Antiviral silencing:** *Ty-1* and *Ty-3* are homologous to Arabidopsis *RDR3, 4,* and *5* and act as resistance determinants against the geminivirus tomato yellow leaf curl virus | **44** |
| ***Medicago truncatula*** | **RDR1** | **Antiviral silencing:** used to transformation of the lab strain of *N. benthamiana* to express *MtRDR1* increased resistance to tobamoviruses | **27,29** |
| ***Oryza stativa* (rice)** | **RDR1** | **Virus induced gene silencing** | **45** |
| | **RDR6** | **Antiviral silencing:** Defence against rice stripe virus<br>**Phytohormone regulation**: Modulation of ABA-modulated gene expression | **46**<br>**47** |
| ***Hordeum vulgare* (barley)** | **RDR1, RDR2, RDR6** | Two *RDR1* genes, an *RDR2* and two *RDR6* genes were identified and expression patterns analyzed following biotic and abiotic treatment | **48** |

| | | | |
|---|---|---|---|
| ***Zea mays* (Maize)** | **RDR2** | **RdDM pathway:** Component of the RdDM pathway | **49,50** |
| ***Gossypium hirsutum* (cotton)** | **RDR6** | Expression reported to be enhanced by jasmonates, ABA, GA, ethylene, wounding, reduced temperature (4 ºC) and NaCl | **51** |
| ***Physcomitrella patens* (moss)** | **RDR6** | **Ta-siRNA pathway:** Developmental regulation | **52** |

**Supplementary Table S2** List of primers used and RNAi hairpin construct sequences

| Primer/ sequence number | Primer/sequence name | Primer or hairpin construct sequence (5' to 3') All PCR primers were designed to have an annealing temperature of 60 °C |
|---|---|---|
| 1 | StRDR1 RNAi 1 F | AAAAAGCAGGCTTCCTCGAGTGATATTGTCCAAGTG |
| 2 | StRDR1 RNAi 1 R | AGAAAGCTGGGTTAGATCTTTTCACGTTCTGCATAGTGA |
| 3 | StRDR1 RNAi 2 F | AAAAAGCAGGCTTCCTCGAGTATCTGCTGACTTTG CTC |
| 4 | StRDR1 RNAi 2 R | AGAAAGCTGGGTTAGATCTCTGGATTCATCCAAACATC |
| 5 | 35S F | CTCTATATAAGGAAGTTCAT |
| 6 | RNAi 1c R | CGGAACTGCAAACATAAGCT |
| 7 | RNAi 2 R | TCAGGCATATAACCACATT |
| 8 | Empty vector R | GGGGACAAGTTTGTACAAAAAAGCAGGCT |
| 9 | Cyclophilin RTqPCR F | GGCACTGGAATTCTCTCCAT |
| 10 | Cyclophilin RTqPCR R | AGATGAAGAACTGAGAACCGTTG |
| 11 | StRDR1 RTqPCR F | TCAAAAATCGGCTCTGGTTC |
| 12 | StRDR1 RTqPCR R | ATGCATCCATGCTGTACCAA |
| 13 | PVX CP RTqPCR F | CACCGTCTGAAGCTGAAATG |
| 14 | PVX CP RTqPCR R | TGGCCTTCGTAATCTTCACA |
| 15 | PVY$^{O}$ CP RTqPCR F | TGGATGGGAATGAACAAGTTGA |
| 16 | PVY$^{O}$ CP RTqPCR R | TGCCTAAGGGTTGGTTTTGC |
| 17 | TMV CP RTqPCR F | CAAGCTCGAACTGTCGTTCA |
| 18 | TMV CP RTqPCR R | CGGGTCTAATACCGCATTGT |
| 19 | StRDR6 RTqPCR F | AGAGGCTGAAGCACGCATA |
| 20 | StRDR6 RTqPCR R | TCCATCTGCTCGAAAACATTC |
| 21 | StRDR2 RTqPCR F | GAGGCAGCTTTAGATGTTTTGG |
| 22 | StRDR2 RTqPCR R | CCTTGGAGCAACATCCTCAT |
| 23 | StRDR1 RNAi 1 | TGATATTGTCCAATTGCGGGCGTATGTGGATCACATGGATGG CATAACTTTGAATTTCGGATGTCAGATATCAGATGACAAGTT TGCAGTGTTGGGAAGTACAGAAGTTTCAATTAAATTTGGCAT TGGATTGAAGAAATTTTTTTTCTTTTTATCTAGTGGTTCAGCT GATTATAAACTTCAGCTTTCATATGAAAATATATGGCAGGTT GTGCTCCATCGTCCATATGGTCAAAATGCTCAGTTTCTCCTC ATACAGTTATTTGGTGCTCCTCGGATCTATAAGAGACTTGAA AACTCCTGTTATAGCTTCTTTAAGGAAACTCCTGATGATCAG TGGGTGAGGACGACAGATTTCGCTCCATCTTGGATAGGGCTA TCTTCTAGCTTATGTTTGCAGTTCCGTAGGGGTGTTCATCTTC CAAATTTCCAGGAAAGTTTTTTTCACTATGCAGAACGTGAAA |
| 24 | StRDR1 RNAi 2 | AATATCTGCTGACTTTGCTCGTAGAGTTGCCTCAAAATGTGG CCTTCAATATACACCATCTGCTTTTCAGATTCGTTATGGTGG ATATAAAGGTGTTGTGGCTGTTGATCCATATTCATCAATGAA GTTATCTTTGAGAAAGAGCATGTCGAAATATGAATCAGACA ACAATAAGTTAGATGTCCTTGGGTGGAGCAAATATCAGCCTT GTTATCTTAATCGTCAACTGGTTACTCTCTTGTCTACACTTGG AGTGAAAGATGATGTTCTCGAACAGAAGCAAAAGGAAGCTG TAGATCAGCTTGATGCTATCTTGCATGATTCTTTGAAGGCAC AGGAGGCTTTGGAATTGATGTCTCCTGGAGAAAACACTAAT ATTCTCAAGGCAATGCTAAATTGTGGTTATATGCCTGATGCT GAGCCCTTTCTTTCAATGATGCTGCAAACCTTCCGCGCATCG AAGTTGCTCGATTTGCGGACTAGATCAAGAATATTTATTCCA AATGGAAGAGCAATGATGGGATGTTTGGATGAATCCAG |

## Supplementary Figures

| RDR | locus ID | DMG ID | DMT ID | DMC ID | DMP ID | potato genome consortium annotations |
|---|---|---|---|---|---|---|
| StRDR1a | PGSC0003DMB000000609:127048..137120 | PGSC0003DMG400031261 | **PGSC0003DMT400080295** | **PGSC0003DMC400054475** | **PGSC0003DMP400054475** * | RNA-dependent RNA-polymerase |
| | PGSC0003DMB000000609:127048..137120 | PGSC0003DMG400031261 | PGSC0003DMT400080296 | PGSC0003DMC400054476 | PGSC0003DMP400054476 | RNA-dependent RNA-polymerase |
| StRDR1b | PGSC0003DMB000000609:94864..103436 | PGSC0003DMG400031269 | PGSC0003DMT400080317 | PGSC0003DMC400054486 | PGSC0003DMP400054486 | RNA-dependent RNA-polymerase |
| | PGSC0003DMB000000609:94864..103436 | PGSC0003DMG400031269 | **PGSC0003DMT400080319** | **PGSC0003DMC400054487** | **PGSC0003DMP400054487** * | RNA-dependent RNA-polymerase |
| StRDR3a | PGSC0003DMB000000005:344441..350234 | PGSC0003DMG400015653 | PGSC0003DMT400040429 | PGSC0003DMC400027425 | PGSC0003DMP400027425 | RNA-dependent RNA polymerase family protein |
| | PGSC0003DMB000000005:344441..350234 | PGSC0003DMG400015653 | PGSC0003DMT400040429 | PGSC0003DMC400027425 | PGSC0003DMP400027425 | RNA-dependent RNA polymerase family protein |
| | PGSC0003DMB000000005:344441..350234 | PGSC0003DMG400015653 | PGSC0003DMT400040431 | PGSC0003DMC400027426 | PGSC0003DMP400027426 | RNA-dependent RNA polymerase family protein |
| | PGSC0003DMB000000005:344441..350234 | PGSC0003DMG400015653 | PGSC0003DMT400040432 | PGSC0003DMC400027427 | PGSC0003DMP400027427 | RNA-dependent RNA polymerase family protein |
| | PGSC0003DMB000000005:344441..350234 | PGSC0003DMG400015653 | **PGSC0003DMT400040433** | **PGSC0003DMC400027428** | **PGSC0003DMP400027428** * | RNA-dependent RNA polymerase family protein |
| | PGSC0003DMB000000005:344441..350234 | PGSC0003DMG400015653 | PGSC0003DMT400040434 | PGSC0003DMC400027429 | PGSC0003DMP400027429 | RNA-dependent RNA polymerase family protein |
| | PGSC0003DMB000000005:344441..350234 | PGSC0003DMG400015653 | PGSC0003DMT400040435 | PGSC0003DMC400027430 | PGSC0003DMP400027430 | RNA-dependent RNA polymerase family protein |
| | PGSC0003DMB000000005:344441..350234 | PGSC0003DMG400015653 | PGSC0003DMT400040436 | PGSC0003DMC400027431 | PGSC0003DMP400027431 | RNA-dependent RNA polymerase family protein |
| StRDR3b | PGSC0003DMB000000155:608365..615653 | PGSC0003DMG401000361 | PGSC0003DMT400000971 | PGSC0003DMC400000713 | PGSC0003DMP400000713 | RNA-dependent RNA polymerase family protein |
| | PGSC0003DMB000000155:608365..615653 | PGSC0003DMG401000361 | PGSC0003DMT400000972 | PGSC0003DMC400000714 | PGSC0003DMP400000714 | RNA-dependent RNA polymerase family protein |
| | PGSC0003DMB000000155:608365..615653 | PGSC0003DMG401000361 | **PGSC0003DMT400000975** | **PGSC0003DMC400000717** | **PGSC0003DMP400000717** * | RNA-dependent RNA polymerase family protein |
| | PGSC0003DMB000000155:608365..615653 | PGSC0003DMG401000361 | PGSC0003DMT400000976 | PGSC0003DMC400000718 | PGSC0003DMP400000718 | RNA-dependent RNA polymerase family protein |
| StRDR3c | PGSC0003DMB000000155:602561..608906 | PGSC0003DMG402000361 | PGSC0003DMT400000974 | PGSC0003DMC400000716 | PGSC0003DMP400000716 | RNA-dependent RNA polymerase family protein |
| | PGSC0003DMB000000155:602561..608906 | PGSC0003DMG402000361 | **PGSC0003DMT400000981** | **PGSC0003DMC400000723** | **PGSC0003DMP400000723** * | RNA-dependent RNA polymerase family protein |
| StRDR6a | PGSC0003DMB000000086:1648259..1654303 | PGSC0003DMG400023601 | **PGSC0003DMT400060673** | **PGSC0003DMC400040837** | **PGSC0003DMP400040837** * | RNA-dependent RNA polymerase 6 |
| StRDR6b | PGSC0003DMB000000494:169979..171250 | PGSC0003DMG400030858 | **PGSC0003DMT400079275** | **PGSC0003DMC400053753** | **PGSC0003DMP400053753** * | RNA-dependent RNA polymerase 6 |
| StRDR6c | PGSC0003DMB000000494:171333..173983 | PGSC0003DMG400030859 | **PGSC0003DMT400079279** | **PGSC0003DMC400053754** | **PGSC0003DMP400053754** * | RNA-dependent RNA polymerase |

Supplementary Figure S1

**Supplementary Figure S1 | Identified potato RDRs from the Potato Genome Sequencing Consortium (PGSC).** Potato *RDR* genes were identified from the PGSC data sequenced from a double monoploid (DM) potato clone *Solanum tuberosum*, group Phureja. The potato RDRs are listed with the PGSC identification numbers for the locus, gene (DMG), transcripts (DMT), coding-sequences (DMC), proteins (DMP) and PGSC annotations. The RDRs are named according to their similarity with other known plant RDRs. Non-redundant proteins are marked with an asterisk. Shading of RDR3a and 3c sequences was added to aid readability.

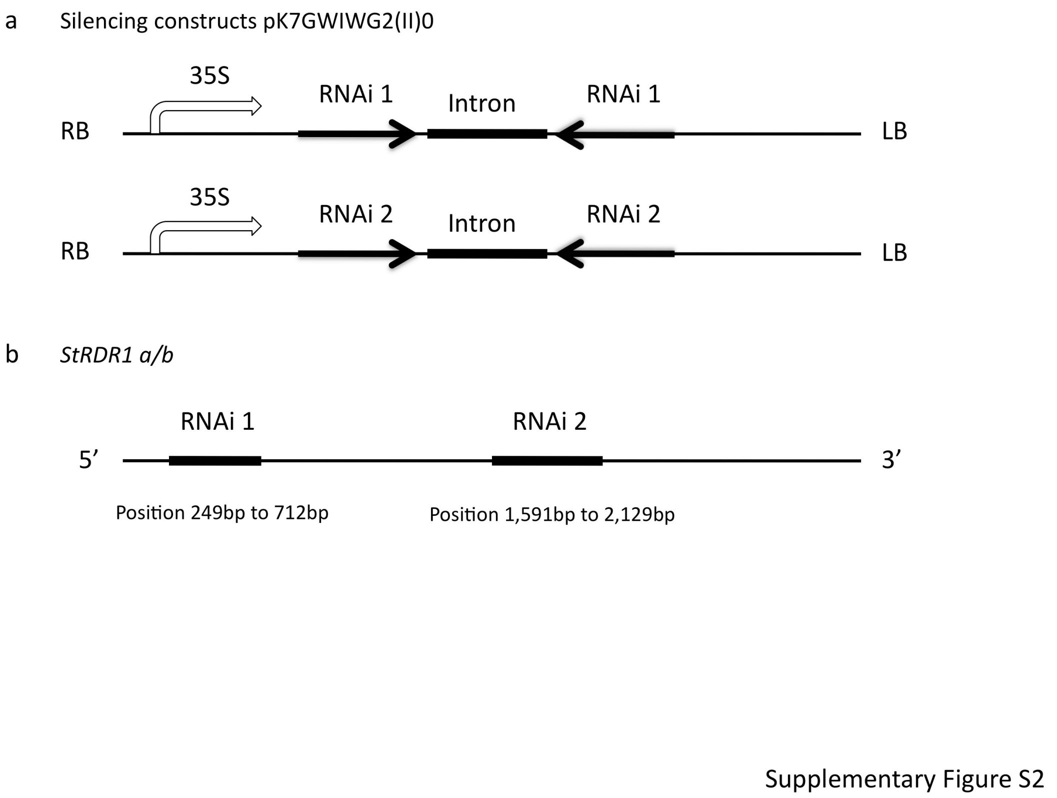


**Supplementary Figure S2 | Diagram of silencing constructs.** Panel a shows the position of the first and the second RNAi sequences between the right border (RB) and the left border (LB) within the silencing construct pK7GWIWG2(II)0 (ref. 53). The sequences are positioned in opposite orientation either side of an intron to produce a hairpin when expressed by the 35S promoter. Panel b illustrates the position of the first and second RNAi sequences on the potato *RDR1a and 1b* genes.

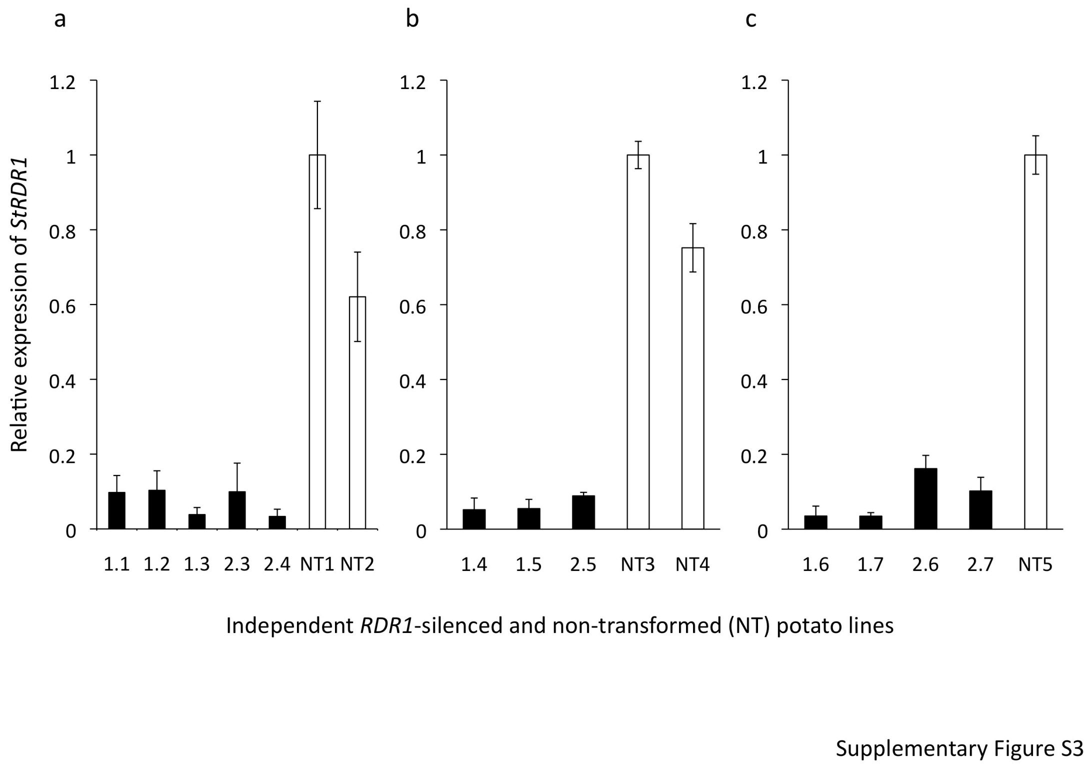


**Supplementary Figure S3 | RTqPCR analysis of *StRDR1* expression in transformed and non-transformed (NT) Pentland Dell potato plants.** RTqPCR analysis of *RDR1* expression was conducted over three experiments shown here in a, b and c. Each experiment included at least one non-transformed (NT) plant as the reference sample and plants carrying each of the RNAi constructs used to silence *RDR1* (lines transformed with the first and second RNAi construct begin with the number one and two, respectively). *Cyclophilin* was used as the reference gene. RNAi construct 1 lines 1.3 and 2.5 were selected for use in the subsequent experiments. The selected lines have significantly lower levels of *StRDR1* expression than the non-transformed lines (Student's *t*-test: *StRDR1* expression of 1.3 in comparison to NT2 $p = 0.0014$; *StRDR1* expression of 2.5 in comparison to NT4 $p = 0.0026$). Error bars represent standard error of the mean.

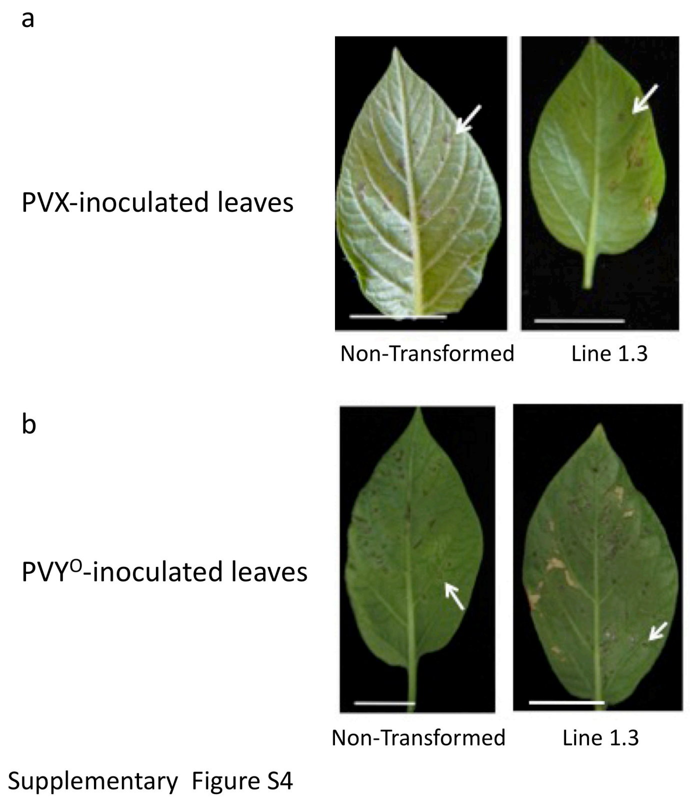


**Supplementary Figure S4 | Appearance of hypersensitive response (HR) lesions on non-transformed and *StRDR1*-depleted transgenic line 1.3 plants.** Potato (background: potato cv. Pentland Dell) 10 days after inoculation with PVX (a) and 7 days post-inoculation with PVY (strain O). Arrows indicate positions of typical HR lesions. The brown areas on the PVY-inoculated Line 1.3 leaf are due to inoculation damage.

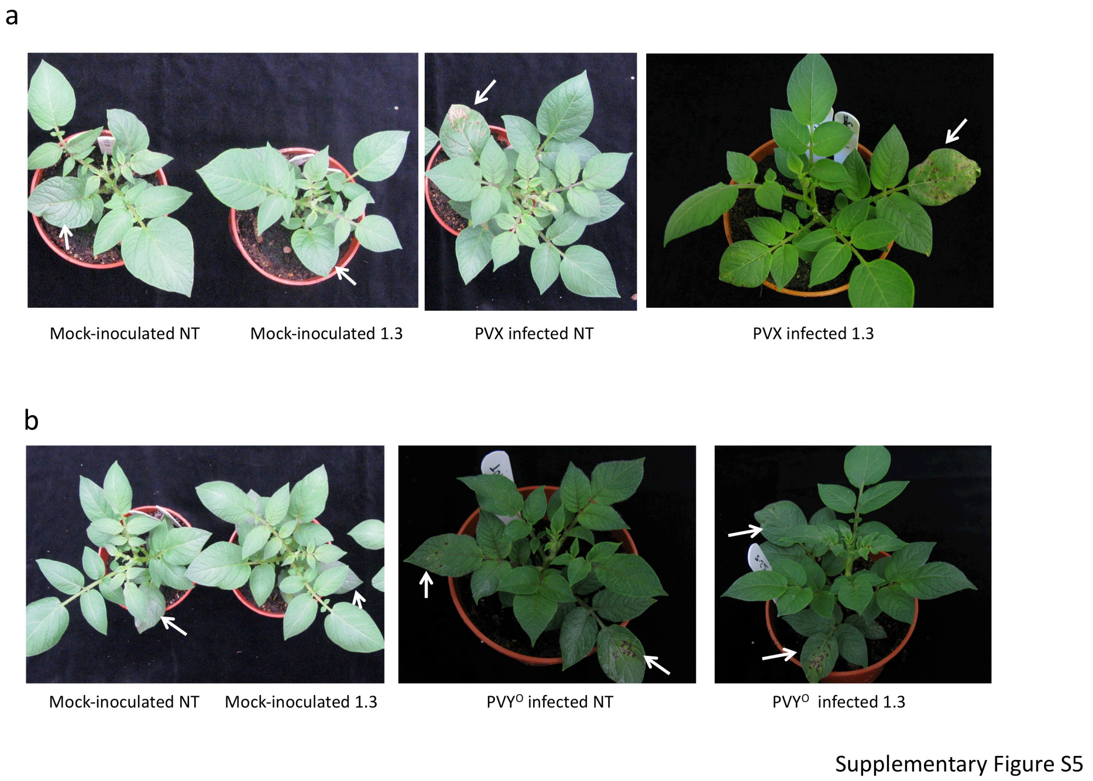


**Supplementary Figure S5 | Responses of potato plants to PVX and PVY$^{O}$.** Appearance of non-transformed (NT) and *StRDR1*-depleted transgenic line 1.3 plants (background: potato cv. Pentland Dell) 10 days after mock-inoculation or inoculation with (a) PVX or (b) PVY (strain O). Inoculated leaves or virus-inoculated leaves are indicated by arrows.

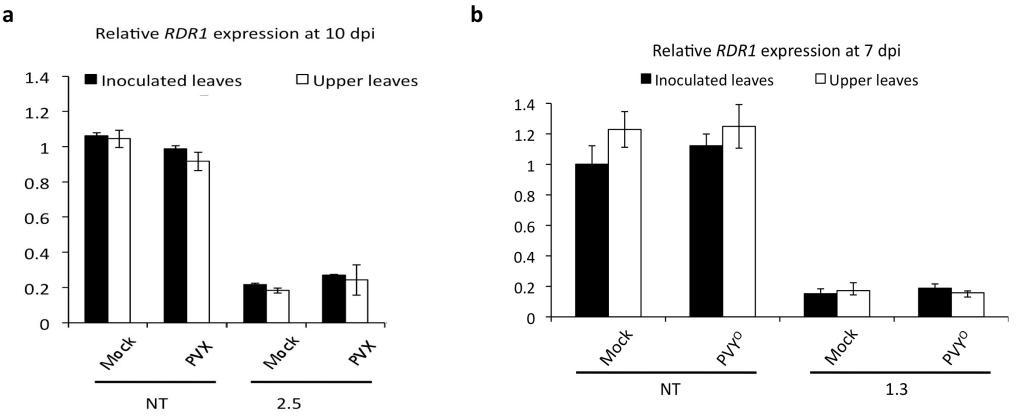


Supplementary Figure S6

**Supplementary Figure S6 | *StRDR1* expression following PVX and PVY$^{O}$ infection**

The relative expression of *RDR1* in mock-inoculated, PVX-infected (a) or PVY$^{O}$-infected plants at 10 and 7 dpi, respectively. RTqPCR was used to analyse *StRDR1* accumulation in directly inoculated and non-inoculated upper leaves of non-transformed (NT) and transgenic lines 1.3 and 2.5 carrying hairpin constructs to inhibit expression of *StRDR1*. Error bars represent standard error of the mean.

**Supplementary Material References**


1. Yu, D., Fan, B., MacFarlane, S.A., Chen, Z. Analysis of the involvement of an inducible *Arabidopsis* RNA-dependent RNA polymerase in antiviral defense. *Molec. Plant-Microbe Interact.* **16,** 206–216 (2003).
2. Donaire, L., Barajas, D., Martínez-García, B., Martínez-Priego, L., Pagán, I. & Llave, C. Structural and genetic requirements for the biogenesis of *Tobacco rattle virus*-derived small interfering RNAs. *J. Virol.* **82,** 5167–5177 (2008).
3. Qi, X., Bao, F.S. & Xie, Z. Small RNA deep sequencing reveals role for *Arabidopsis thaliana* RNA-dependent RNA polymerases in viral siRNA biogenesis. *PLoS One* **4,** e4971 (2009).
4. Garcia-Ruiz, H., Takeda, A., Chapman, E.J., Sullivan, C.M., Fahlgren, N., Brempelis, K.J. & Carrington, J.C. *Arabidopsis* RNA-dependent RNA polymerases and dicer-like proteins in antiviral defense and small interfering RNA biogenesis during *Turnip mosaic virus* infection. *Plant Cell* **22,** 481–496 (2010).
5. Wang, X. *et al.* RNAi-mediated viral immunity requires amplification of virus-derived siRNAs in *Arabidopsis thaliana*. *Proc. Nat. Acad. Sci. USA* **107**, 484–489 (2010).
6. Dalmay, T., Hamilton, A., Rudd, S., Angell, S. & Baulcombe, D.C. An RNA-dependent RNA polymerase gene in *Arabidopsis* is required for posttranscriptional gene silencing mediated by a transgene but not by a virus. *Cell* **101,** 543–553 (2000).
7. Mourrain, P., Béclin, C., Elmayan, T., Feuerbach, F., Godon, C., Morel, J.B., Jouette, D., Lacombe, A.M., Nikic, S., Picault, N., Rémoué, K., Sanial, M., Vo, T.A. & Vaucheret, H. *Arabidopsis SGS2* and *SGS3* genes are required for posttranscriptional gene silencing and natural virus resistance. *Cell* **101,** 533–542 (2000).
8. Muangsan, N., Beclin, C., Vaucheret, H. & Robertson, D. Geminivirus VIGS of endogenous genes requires SGS2/SDE1 and SGS3 and defines a new branch in the genetic pathway for silencing in plants. *Plant J.* **38,** 1004–1014 (2004).
9. Vazquez, F. *et al.* Endogenous *trans*-acting siRNAs regulate the accumulation of *Arabidopsis* mRNAs. *Molec. Cell* **16**, 69–79 (2004).
10. Peragine, A., Yoshikawa, M., Wu, G., Albrecht, H.L. & Poethig, R.S. *SGS3* and *SGS2/SDE1/RDR6* are required for juvenile development and the production of *trans*-acting siRNAs in *Arabidopsis*. *Genes Dev.* **18**, 2368–2379 (2004).
11. Wu, G. & Poethig, R.S. Temporal regulation of shoot development in *Arabidopsis thaliana* by miR156 and its target SPL3. *Development* **133**, 3539–3547 (2006).
12. Li, H. *et al.* The putative RNA-dependent RNA polymerase *RDR6* acts synergistically with *ASYMMETRIC LEAVES1* and *2* to repress *BREVIPEDICELLUS* and MicroRNA165 / 166 in Arabidopsis leaf development. *Plant Cell* **17**, 2157–2171 (2005).
13. Xu, L. *et al.* Genetic interaction between the AS1-AS2 and RDR6-SGS3-AGO7 pathways for leaf morphogenesis. *Plant Cell Physiol.* **47**, 853–863 (2006).
14. Yoon, E.K. *et al.* Auxin regulation of the *microRNA390*-dependent transacting small interfering RNA pathway in *Arabidopsis* lateral root development. *Nucleic Acids Res.* **38**, 1382–1391 (2010).
15. Fahlgren, N. *et al.* Regulation of AUXIN RESPONSE FACTOR3 by TAS3 ta-siRNA affects developmental timing and patterning in Arabidopsis. *Curr.*

*Biol.***16**, 939-44 (2006).
16. Borsani, O., Zhu, J., Verslues, P.E., Sunkar R. & Zhu, J.K. Endogenous siRNAs derived from a pair of natural *cis*-antisense transcripts regulate salt tolerance in Arabidopsis. *Cell* **123**, 1279-1291 (2005).
17. Katiyar-Agarwal, S. *et al.* A pathogen-inducible endogenous siRNA in plant immunity. *Proc. Nat. Acad. Sci. USA* **103**, 18002–18007 (2006).
18. Xie, Z. et al. Genetic and functional diversification of small RNA pathways in plants. *PLoS Biol.* **2**, E104 (2004).
19. Pontes, O. *et al.* The *Arabidopsis* chromatin-modifying nuclear siRNA pathway involves a nucleolar RNA processing center. *Cell* **126**, 79–92 (2006).
20. Lu, C. *et al.* MicroRNAs and other small RNAs enriched in the *Arabidopsis* RNA-dependent RNA polymerase-2 mutant. *Genome Res.* **16**, 1276–1288 (2006).
21. Kasschau, K.D. *et al.* Genome-wide profiling and analysis of *Arabidopsis* siRNAs. *PLoS Biol.* **5**, e57 (2007).
22. Haag, J.R. *et al. In vitro* transcription activities of Pol IV, Pol V , and RDR2 reveal coupling of Pol IV and RDR2 for dsRNA synthesis in plant RNA silencing. *Molec. Cell* **48**, 811–818 (2012).
23. Smith, L.M. *et al.* An SNF2 protein associated with nuclear RNA silencing and the spread of a silencing signal between cells in *Arabidopsis*. *Plant Cell* **19**, 1507–1521 (2007).
24. Brosnan, C.A. *et al.* Nuclear gene silencing directs reception of long-distance mRNA silencing in Arabidopsis. *Proc. Natl. Acad. Sci. USA* **104**, 14741-14746 (2007).
25. Xie, Z., Fan, B., Chen, C. & Chen, Z. An important role of an inducible RNA-dependent RNA polymerase in plant antiviral defense. *Proc. Nat. Acad. Sci. USA* 98, 6516–6521 (2001).
26. Rakhshandehroo, F., Takeshita, M., Squires, J. & Palukaitis, P. The influence of RNA-dependent RNA polymerase 1 on *Potato virus Y* infection and on other antiviral response genes. *Molec. Plant-Microbe Interact.* **22,** 1312–1318 (2009).
27. Yang, S.-J., Carter, S.A., Cole, A.B., Cheng, N.-H. & Nelson, R.S. A natural variant of a host RNA-dependent RNA polymerase is associated with increased susceptibility to viruses by *Nicotiana benthamiana*. *Proc. Nat. Acad. Sci. USA* **101,** 6297–6302 (2004).
28. Ying, X. *et al.* RNA-dependent RNA polymerase 1 from *Nicotiana tabacum* suppresses RNA silencing and enhances viral infection in *Nicotiana benthamiana*. *Plant Cell* **22**, 1358–1372 (2010).
29. Wylie, S.J. *et al.* Differential responses to virus challenge of laboratory and wild accessions of Australian species of *Nicotiana*, and comparative analysis of *RDR1* gene sequences. *PLoS One* **10,** e0121787 (2015).
30. Schwach, F., Vaistij, F.E, Jones, L. & Baulcombe, D.C. An RNA-dependent RNA polymerase prevents meristem invasion by potato virus X and is required for the activity but not the production of a systemic silencing signal. *Plant Physiol.* **138,** 1842–1852 (2005).
31. Qu, F. *et al.* RDR6 has a broad-spectrum but temperature-dependent antiviral defense role in *Nicotiana benthamiana. J. Virol.* **79**, 15209–15217 (2005).
32. Vaistij, F.E., Jones L., & Baulcombe, D.C. Spreading of RNA targeting and DNA methylation in RNA silencing requires transcription of the target gene and a putative RNA-dependent RNA polymerase. *Plant Cell* **14**, 857-67 (2002).
33. Vaistij, F.E. & Jones, L. Compromised virus-induced gene silencing in RDR6-

deficient plants. *Plant Physiol.* **149**, 1399–1407 (2009).

34. Di Serio, F., Martínez de Alba, A.-E., Navarro, B., Gisel, A. & Flores, R. RNA-dependent RNA polymerase 6 delays accumulation and precludes meristem invasion of a viroid that replicates in the nucleus. *J. Virol.* **84**, 2477–2489 (2010).
35. Gómez, G., Martínez, G. & Pallás, V.Viroid-induced symptoms in Nicotiana benthamiana plants are dependent on RDR6 activity. *Plant Physiol.* **148**, 414-23 (2008).
36. Pandey, S.P. & Baldwin, I.T. RNA-directed RNA polymerase 1 (RdR1) mediates the resistance of *Nicotiana attenuata* to herbivore attack in nature. *Plant J.* **50,** 40–53 (2007).
37. Pandey, S.P., Shahi, P., Gase, K. & Baldwin, I.T. Herbivory-induced changes in the small-RNA transcriptome and phytohormone signalling in *Nicotiana attenuata. Proc. Nat. Acad. Sci. USA* **105**, 4559–4564 (2008).
38. Pandey, S.P. & Baldwin, I.T. Silencing RNA-directed RNA polymerase 2 increases the susceptibility of *Nicotiana attenuata* to UV in the field and in the glasshouse. *Plant J.* **54**, 845-862 (2008).
39. Pandey, S.P., Gaquerel, E., Gase, K. & Baldwin, I.T. *RNA-directed RNA polymerase3* from *Nicotiana attenuata* is required for competitive growth in natural environments. *Plant Physiol.* **147**, 1212–1224 (2008).
40. Liu, Y., Gao, Q., Wu, B., Ai, T. & Guo, X. *NgRDR1*, an RNA-dependent RNA polymerase isolated from *Nicotiana glutinosa*, was involved in biotic and abiotic stresses. *Plant Physiol. Biochem.* **47,** 359–368 (2009).
41. Yang, H., Wang, M., Gao, Z., Zhu, C. & Guo, X. Isolation of a novel RNA-dependent RNA polymerase 6 from *Nicotiana glutinosa*, *NgRDR6*, and analysis of its response to biotic and abiotic stresses. *Molec. Biol. Rep.* **38,** 929–937 (2011).
42. Kuang, H. *et al.* Identification of miniature inverted-repeat transposable elements (MITEs) and biogenesis of their siRNAs in the Solanaceae: new functional implications for MITEs. *Genome Res.* **19**, 42–56 (2009).
43. Schiebel, W. *et al.* Isolation of an RNA-directed RNA polymerase-specific cDNA clone from tomato. *Plant Cell* **10**, 2087–2101 (1998).
44. Verlaan, M.G. *et al*. The tomato yellow leaf curl virus resistance genes *Ty-1* and *Ty-3* are allelic and code for DFDGD-class RNA–dependent RNA polymerases. *PLoS Genetics* **9**, e1003399 (2013).
45. Chen, H. *et al.* Analysis of rice *RNA-dependent RNA polymerase 1* (*OsRDR1*) in virus-mediated RNA silencing after particle bombardment. *J. Gen. Plant Pathol.* **76,** 152–160 (2010).
46. Jiang, L. *et al.* RNA-dependent RNA polymerase 6 of rice (*Oryza sativa*) plays role in host defense against negative-strand RNA virus, *Rice stripe virus*. *Virus Res.* **163,** 512–519 (2012).
47. Yang, J.H. *et al.* Phytohormone abscisic acid control RNA-dependent RNA polymerase 6 gene expression and post-transcriptional gene silencing in rice cells. *Nucleic Acids Res.* **36**,1220–1226 (2008).
48. Madsen, C.T. *et al.* Identification and characterization of barley RNA-directed RNA polymerases. *Biochimica et Biophysica Acta* **1789,** 375–385 (2009).
49. Jia, Y. *et al.* Loss of RNA-dependent RNA polymerase 2 (RDR2) function causes widespread and unexpected changes in the expression of transposons, genes, and 24-nt small RNAs. *PLoS Genet.* **5**, e1000737 (2009).
50. Nobuta, K. *et al.* Distinct size distribution of endogenous siRNAs in maize:

Evidence from deep sequencing in the *mop1-1* mutant. *Proc. Nat. Acad. Sci. USA* **105**, 14958–14963 (2008).
51. Wang, M., Li, S., Yang, H., Gao, Z., Wu, C. & Guo, X. Characterization and functional analysis of *GhRDR6*, a novel *RDR6* gene from cotton (*Gossypium hirsutum* L.). *Biosci. Rep.* **32**, 139–151 (2012).
52. Talmor-Neiman M. *et al.* Identification of *trans*-acting siRNAs in moss and an RNA-dependent RNA polymerase required for their biogenesis. *Plant J.* **48**, 511–521 (2006).
53. Karimi, M., Inzé, D. & Depicker, A. GATEWAY™ vectors for *Agrobacterium*-mediated plant transformation. *Trends Plant Sci.* **7**, 193–195 (2002).
